# Exploring Cardiorespiratory Resilience and Mobility as Indicators of Physical Fitness Under Individualised Therapy Intervals in Obese Dogs

**DOI:** 10.3390/ani16040678

**Published:** 2026-02-21

**Authors:** Paula Welter, Oliver Harms, Holger A. Volk, Julia D. Kschonek, Ammelie Godglück, Christian Visscher, Volker Wilke

**Affiliations:** 1 Clinic for Small Animals, University of Veterinary Medicine Hannover, Foundation, Bünteweg 9, 30559 Hannover, Germany; dr.o.harms@googlemail.com (O.H.); holger.volk@tiho-hannover.de (H.A.V.); julia.dorothee.kschonek@tiho-hannover.de (J.D.K.); 2 Fachzentrum für Kleintiermedizin Langenhagen, Bayernstraße 17, 30855 Langenhagen, Germany; 3 Institute for Animal Nutrition, University of Veterinary Medicine Hannover, Foundation, Bischofsholer Damm 15, 30173 Hannover, Germany; ammelie.godglueck@tiho-hannover.de (A.G.); christian.visscher@tiho-hannover.de (C.V.); wilke@tierklinik-sarstedt.de (V.W.); 4 Tierklinik Sarstedt, Wenderter Straße 7, 31157 Sarstedt, Germany

**Keywords:** obesity, body condition score, physical fitness, gait analysis, fitness test

## Abstract

Obesity in dogs is an increasing health and animal welfare problem as it is associated with numerous secondary diseases and reduced life span. While the causes and consequences of obesity are well documented, the change in cardiorespiratory resilience and mobility parameters in addition to weight loss has rarely been studied. The aim of this study was to analyse the change effects during individualised obesity therapy intervals in accordance with the current state of research on canine obesity and on physical fitness. In a prospective single-arm, non-randomised study without a control arm, 13 obese dogs (Body condition score (BCS) ≥ 7/9) were included. To assess mobility, a submaximal fitness test and gait analysis on a treadmill were performed before and after therapy. The dogs reduced their body weight by 15.26 ± 4.99%. After weight loss, the dogs showed statistically significantly lower heart rates and lactate values, as well as higher pCO_2_ and bicarbonate values as indicators of cardiorespiratory resilience. Concerning parameters of mobility, statistically significant changes in the absolute forces and the asymmetrical force distribution were found. The force distribution between the thoracic and pelvic limbs showed a shift towards the pelvic limbs and the symmetry index of the pelvic limbs was lower. The data collected in this study showed how body weight in obese dogs, but also physical fitness parameters change during therapy intervals. The results of this study provide effect sizes to implement standardised submaximal fitness tests and gait analysis as tools for objective monitoring of therapy progress in future studies.

## Simple Summary

Obesity is one of the most common health problems in pet dogs and can greatly reduce their quality of life, increase the risk of many diseases, and shorten their lifespan. This study examined whether helping overweight dogs lose weight would also improve their physical fitness, meaning how well their bodies cope with activity and exercise. Thirteen overweight dogs took part in a weight-loss therapy that provided an individually calculated low-energy diet along with regular support and guidance for the owners. Before and after the therapy, the dogs completed a treadmill test, and their movement was measured to assess how their bodies responded to exercise. On average, the dogs lost a meaningful amount of body weight and showed clear improvements during the fitness test, such as lower heart rates and lower levels of physical stress after activity. These results show that weight loss not only makes dogs leaner but also helps them move more easily and cope better with exercise, which is valuable for their long-term well-being.

## 1. Introduction

Obesity is the most prevalent diet-related disorder in dogs, with reported prevalence rates reaching up to 65% in certain populations [1]. Beyond compromising the animals’ well-being [2], obesity is associated with a reduced life expectancy that varies by breed and can be shortened by up to 3 years [3]. Moreover, overweight dogs are at increased risk for a wide range of secondary conditions, including endocrine and metabolic diseases, orthopaedic disorders, cardiorespiratory impairments, and urinary tract disorders [4,5]. In addition, overweight dogs are more frequently affected by certain types of cancer [6]. Given the profound impact of obesity on canine health, it must be addressed not only from a medical but also from an animal welfare perspective [5].

Relevant factors that determine the success of obesity therapy have been evaluated [5,7,8,9,10]. Obesity in dogs is generally defined as body weight exceeding 10% above the ideal body weight for an individual. Overweight is associated with an increased formation of body fat, which can be reliably assessed in practice using the body condition score (BCS) [1,11,12,13]. The primary goal of obesity therapy is to achieve sustainable weight loss while preserving muscle mass, thereby improving body composition and overall health [8,10,14,15]. A common monitoring approach during therapy intervals is to evaluate the dog’s weight, the BCS, and muscle condition score (MCS) [10,14,15,16]. However, these parameters primarily reflect body composition and do not fully capture systemic health status or functional capacity, potentially overlooking important aspects of the dog’s overall well-being.

With the aim of extending the common monitoring approaches, the present study investigated the changes in objective physical fitness parameters of overweight dogs, specifically cardiorespiratory resilience and mobility. These parameters are of particular relevance to the dog’s quality of life and long-term health, as they reflect functional capacity and physical fitness [1,5,17]. The assessment of these parameters through standardised gait analysis, blood biomarkers, and clinical diagnostics provides a robust, objective, and reproducible means of evaluating therapeutic outcomes in daily veterinary practice. Beyond characterising the kind and magnitude of parameter change within therapy intervals, this study implemented individualised treatment plans based on current scientific guidelines [7,8,9,10], thereby assessing how cardiorespiratory and mobility parameters evolve within a pragmatic, real-world clinical setting.

## 2. Materials and Methods

### 2.1. Study Design and Population

The goal of the study is to explore how cardiorespiratory and mobility parameters of obese dogs change during individualised therapy intervals. The study was designed as a single-arm, non-randomised trial without a control group. The primary objective was to prospectively assess outcomes under individualised, real-life conditions rather than to compare standardised interventions. The generated, descriptive effect size estimates (estimands) shall inform the design of future randomised trials and hypothesis-driven research appropriate in future study design [18,19].

The inclusion of a control arm was not deemed appropriate, as withholding standardised weight reduction therapy to reduce obesity-related comorbidities and improve quality of life would raise ethical concerns. Moreover, in the absence of intervention, the expected mean change in body weight and mobility in obese dogs is assumed to be approximately zero over the study period, with deviations primarily attributable to natural inter-individual variability. Although historical or external control data are not available to construct a post hoc comparator, this assumption provides a pragmatic reference for the interpretation of single-arm trial results [20].

The study was conducted between November 2023 and November 2024 at the Clinic for Small Animals of the University of Veterinary Medicine Hanover. The study group consisted of privately owned dogs with manifest obesity, defined as a BCS of at least 7/9, orienting on the definition of obesity in previous studies. Successful body weight loss was defined as a reduction of at least one BCS point from baseline [11,12,13]. Participating dogs were either already patients at the clinic or the owners learned about the opportunity to participate via social media. Hence, the dog’s owners were interested in treating their dogs’ obesity. Otherwise, no selection criteria regarding breed, age, or existing diseases were defined.

### 2.2. Data Collection Steps

Owner’s perspective: At the beginning of the study, the owners were asked to complete a questionnaire on their dogs’ exercise habits and current diet. In addition, they were asked to self-assess their dogs’ BCS and state both their current weight and their estimated target weight. This self-assessment was collected because dog owners often underestimate the nutritional status of their animals, which can make it difficult to determine the need for therapy or its goal [9].

Clinical assessment: On the day of the initial examination, dogs presented starved for at least 12 h. First, a detailed medical history was taken to gather relevant information on the dogs’ state of health and previous history. Second, a general examination, laboratory examination (including haematology, clinical chemistry, and measurement of TSH and T4) [9,21], BCS, and MCS measurements [11,16] were recorded in the patient recording system of the clinic (easyVet, VetZ GmbH).

Physical fitness: The dogs were acclimatised to a treadmill, on which the fitness test and gait analysis were performed. The protocol was established at the Clinic for Small Animals at the University of Veterinary Medicine Hanover in previous studies [22,23,24]. The conduct of the tests is described in more detail further below.

**Figure 1:**
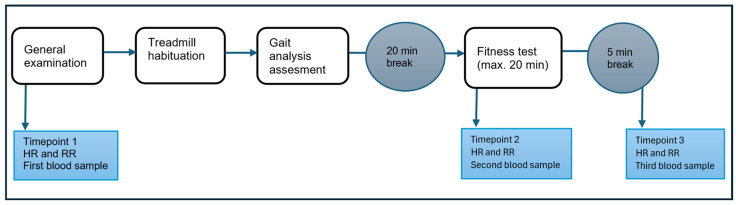
Procedure of the initial and final examinations, including the fitness test and gait analysis. HR = heart rate, RR = respiratory rate.

Based on the results of the initial examination (before therapy), the owners received an individually adapted therapy plan, which was developed together with the Institute of Animal Nutrition, University of Veterinary Medicine Hanover. During the therapy interval, owners regularly monitored their dogs’ weight at home [7,8,9,10,25]. After successful weight loss, a final examination was carried out, in which all measures were recorded again to evaluate the change in mobility and cardiorespiratory parameters. An overview of the data collection steps is provided in Figure 1.

### 2.3. Dietary Treatment

After the initial examination and fitness test, the ideal body weight for a dog was determined based on the BCS and veterinary assessment. The maintenance energy requirement for the ideal body weight was calculated, and individual daily rations were subsequently set to 60–70% of this maintenance energy requirement to promote controlled weight loss. Diet formulation was aligned with the dog’s previous feeding practices and the preferences of the owners.

In total, four raw or home-cooked diets, six commercial dry diets, and three combinations of dry and wet diets were used. All diets were evaluated for energy and nutrient content and adjusted to meet individual requirements. Body weight was monitored at regular intervals of one to two weeks through the study by the owners. Based on these measurements, the energy intake was adapted if necessary to ensure a steady and clinically appropriate weight loss of 1 to 2% of body weight per week.

Once the dogs reached their individual target weights, new rations were calculated to provide the estimated maintenance energy requirement for the ideal body weight, with the aim of maintaining the achieved optimal nutritional status over the long term. A detailed table with further information about the diets (including feeding practice, ME%, protein%, fat%, and fibre%) is attached in the Supplementary Material (Table S1).

### 2.4. Fitness Test

To determine fitness level, the participating dogs completed an individually adapted submaximal fitness test, which has been established in previous studies at the University of Veterinary Medicine Hanover [24,26,27]. The dogs were first familiarised with the treadmill in a habituation phase until they were able to move comfortably without signs of stress or resistance. Then, an individual running speed was determined at which the dogs showed a relaxed trot. A relaxed trot was defined operationally as a symmetrical, rhythmic gait, characterised by a steady head and trunk position, absence of pulling on the leash, and no observable signs of stress. After a break of 20 min, the dogs ran at this individually determined speed for a maximum of 20 min during the exercise phase.

At three timepoints, before the habituation phase, immediately before the actual exercise phase, and after the exercise, the dogs’ heart rate and respiratory rate were assessed. Also, a venous blood sample was taken. Venous blood was collected from the lateral saphenous vein and was analysed immediately after collection (maximally within 10 min) using a Siemens Rapidlab1260 blood gas analyser (Thermo Fisher Scientific, Waltham, MA, USA) at the on-site laboratory. Results were reported at the analyser’s default temperature setting (37 °C). All measurements were performed in the same air-conditioned examination room maintained at approximately 21 °C. Lactate, carbon dioxide partial pressure, oxygen partial pressure, bicarbonate, base excess, and pH value were measured in the blood sample.

If there were signs of fatigue during the exercise phase, such as repeated slowing or stopping, uncoordinated movements, or very heavy panting, the exercise phase was ended prematurely—before the 20 min had elapsed. The individual speed and time of exercise were documented and repeated during the final examination to allow for intra-individual comparison.

### 2.5. Gait Analysis

Gait analysis was performed using a four-section treadmill with integrated force plates (Model 4060-08, Bertec Corporation, Columbus, OH, USA). The plates measure the ground reaction forces of the individual limbs in the three dimensions (x-, y-, and z-axis). The vertical ground reaction force (Fz) was used for the evaluation. The maximum value (peak Fz), the mean value (mean Fz) and the impulse (impulse Fz) were analysed. The data were collected and processed using the Vicon Nexus 1.8.5 software (Vicon Nexus 1.8.5, Vicon Motion Systems Ltd., Oxford, UK).

At the beginning, the dogs were familiarised with the treadmill to ensure that they could move on the treadmill surface without stress or uncertainty. During this time, the individual trotting speed of each dog was determined to ensure a smooth and stable movement for the measurements. The measurement itself was carried out during a phase of trotting that was as steady as possible, avoiding external influences such as sudden changes in direction or posture. For the evaluation of the gait analysis, 10 representative steps from such a stable trotting phase were later manually selected. The selected steps were time-standardised to 100% of the stance phase in order to obtain a meaningful and comparable analysis of the movement dynamics of each dog.

Step selection was performed by a single experienced investigator using the same criteria for all recordings. In addition to the absolute values, the measured forces (e.g., IFz, PFz, and MFz) were also normalised to the body weight of the dogs (later signified by “%”, e.g., “IFz%”). The weight distribution of the force on the thoracic and pelvic limbs was calculated (LD = (Sum of (force) left and right thoracic limb)/(Sum of (force) left and right pelvic limb)) and a symmetry index between the right and left thoracic and pelvic limb were calculated (SI = 100 − ((Fz lower/Fz higher) × 100)) orienting on [22,28,29,30].

### 2.6. Statistical Analysis

First, a descriptive analysis of collected data was performed. To assess the within-subject change in parameters over the therapy interval, a paired *t*-test or Wilcoxon signed-rank-test was conducted. The statistical analysis was performed using the SAS^®^ statistical software, version 9.4M7 (SAS Institute Inc., Cary, NC, USA).

Then, normality of the within-subject differences was assessed using the Shapiro–Wilk test. In case of significant deviation from normality (*p* ≤ 0.05), the Wilcoxon signed-rank test was conducted. In the text, results were reported using the signed-rank statistic “S” and the *p*-value. As a descriptive measure of effect magnitude, the absolute mean (pre–post therapy) difference was reported. Also, Cohen’s *d* is reported to quantify the magnitude of the changes, independent of the statistical test used.

If the normality assumption of differences was not violated (*p* > 0.05), paired *t*-tests were used. Effect sizes were quantified using Cohen’s *d* for paired samples, alongside mean differences, 95% confidence intervals, and *p*-values.

To assess systematic differences in repeated gait measurements (10 selected steps per dog during a stable trotting phase), a linear mixed-effects model with a random intercept for subjects was used to account for the correlation of repeated measurements within individuals. While the estimated effects for the pre–post comparison are reported in the main manuscript, full model specifications and parameter estimates are provided in the Supplementary Materials.

Due to the small sample size, formal inference regarding causal effects was not pursued. Consequently, no correction for multiple testing was applied, as such adjustments are generally less meaningful in exploratory analyses with limited statistical power.

## 3. Results

### 3.1. Study Population and Progress of Therapy

A total of 18 overweight dogs were presented in the initial examination. From this group, 13 dogs could be re-examined after obesity therapy and were included in the study. The contact with owners of the five excluded dogs was lost during the therapy or the owners chose not to continue participating in the study, so these dogs were not presented in the final examination. The reasons for withdrawal were primarily logistical and unrelated to the intervention itself, including changes in owner availability or personal circumstances (n = 3) and unrelated health or behavioural issues that precluded continued participation (n = 2). None of the dogs were withdrawn due to adverse effects or intolerance to the intervention.

The breeds of included dogs included a Great Swiss Mountain Dog, four Labrador Retrievers, three Australian Shepherds, a German Shepherd, a Mini American Shepherd, a Beagle, and two mixed breeds. Most dogs were neutered females (38.46%) and neutered male dogs (30.77%). More intact females (23.08%) than males (7.69% = one dog) participated. The dogs were on average 7 years old (min. 4, max. 10 years).

The median BCS before therapy was 7/9 and the maximum BCS of 8/9 was recorded in five dogs. The majority of dogs had a normal muscle condition score (MSC) before therapy (69.23%), with the maximum of ‘moderate loss’ in one dog (7.69%). A detailed table with further information about the study population is attached in the Supplementary Material (Tables S2 and S3).

During therapy, the dogs’ BCS decreased statistically significantly, on average about 2 score points (paired Wilcoxon signed-rank test, |S| = 45.5, *p* = 0.0002). Similarly, statistically significant changes in weight in kilograms (mean difference 4.87 kg) and a mean weight change of 15.33% over time were recorded (paired *t*-test, *p* < 0.0001 for both). Comparing the effect sizes, Cohen’s d was highest for the BCS, followed closely by weight loss in percentage. The MCS change during therapy showed no statistically significant change (|S| = 5, *p* = 0.125) (Table 1).

### 3.2. Fitness Test

The duration of the fitness test for the 13 dogs was 16.47 ± 4.45 min. In 7 of the 13 dogs (53.8%), the test was discontinued prior to the completion of the 20 min period due to signs of exhaustion. The speed at which the fitness test was performed was 1.81 ± 0.42 m/s.

All dogs exhibited panting after exercise during both the initial and final examination, rendering the analysis of the respiratory rate impossible. Four parameters after exercise (timepoint 3, “t3”) were statistically significant, and base excess (“BE”) slightly missed statistical significance (paired *t*-Test, t = 2.05, *p* = 0.063) (Table 2).

One of the parameters that showed a statistically significant change during therapy was the heart rate (HR; t = −3.52, *p* < 0.01). After exercise, mean heart rate decreased from 137.39 beats/min before therapy to 110.92 beats/min after therapy.

Venous blood lactate concentration measured after exercise decreased significantly from 2.25 mmol/L before therapy to 1.78 mmol/L after weight loss (t = −2.29, *p* < 0.05). Similarly, post-exercise partial pressure of carbon dioxide (pCO_2_) increased from 23.88 mmHg to 27.26 mmHg after therapy (t = 2.26, *p* < 0.05). Blood bicarbonate concentration (HCO_3_^−^) measured after exercise increased from 17.91 mmol/L to 19.66 mmol/L following the therapy (t = 2.55, *p* < 0.05).

In terms of effect size, the change in heart rate (Cohen’s *d* = −0.98) and bicarbonate concentration (Cohen’s *d* = 0.71) both indicated large effects. Descriptive statistics and the complete results of statistical tests are provided in the Supplementary Materials (Tables S3 and S4).

### 3.3. Gait Analysis

The absolute vertical ground reaction forces, PFz (peak), MFz (mean), and IFz (impulse), show a statistically significant difference (decrease) before and after therapy. The relative vertical ground reaction forces, PFz (peak), MFz (mean), and IFz (impulse), normalised to body weight show a statistically significant change (increase) in all limbs except for the MFz and IFz of the right thoracic limb after therapy. An excerpt of statistically significant estimates of absolute force changes is provided in Table 3.

Concerning the change in IFz thoracic (left/right) and pelvic limbs (left/right), mean values show a shift towards the pelvic limbs in both absolute and relative forces. The percentage IFz of the thoracic limbs was 61.62% before and 60.37% after therapy. The IFz of the pelvic limb was 38.38% before and 39.63% after therapy. Further descriptive statistics and test results of changed load distribution and model statistics are provided in the Supplementary Materials (Tables S3 and S5–S7).

## 4. Discussion

This single-arm, non-randomised trial without a control group explored the changes in mobility and cardiorespiratory resilience parameters during therapy. The study was performed with overweighted dogs showing an average weight loss of 15% and a reduction in BCS by two score points (from 7.39 to 5.39).

Physical fitness related to submaximal exercise and cardiorespiratory response improved significantly after weight loss. After weight loss, dogs’ heart rate post-exercise (timepoint 3) dropped significantly, by 19%, lactate decreased by 21%, pCO_2_ increased by 14%, and bicarbonate increased by 10%. Other parameters examined, such as pO_2_, base excess, and pH, showed no significant differences before and after therapy. A similar pattern of changes was described in the study by Willen et al. (2021), which investigated differences in physical fitness between young and old beagles. Younger animals showed lower lactate levels and heart rates after exercise [24]. The changes observed in the present study are similar, especially regarding heart rates and lactate levels after exercise and therapy.

In contrast to our study results, Wall et al. (2018) found no significant differences in heart rate or metabolic parameters immediately after exercise in dogs with pre-symptomatic mitral valve disease compared to healthy control animals [23]. The fact that the present study was able to demonstrate significant changes despite submaximal exercise suggests that obesity is a relevant factor influencing exercise tolerance independently of underlying cardiovascular disease. Additional evidence is provided by the work of Türkcü et al. (2023) and Mach et al. (2022), who also used the fitness test for brachycephalic dog breeds. They showed a statistically significant exercise tolerance reduction, even in otherwise healthy dogs, indicating the importance of breed-specific morphological limitations [26,27]. The results of this study illustrate physiological exercise tolerance improvements during therapy intervals. Overall, these observations underscore the suitability of the submaximal fitness test used as an instrument for assessing physical fitness in overweight dogs, as well as the value of using cardiorespiratory parameters to observe improvement of functional capacity. The significant improvements after therapy show that not only external appearance but also functional benefits in terms of physical resilience can be observed.

Given the submaximal testing protocol, the results do not allow conclusions to be drawn regarding maximal exercise tolerance. In the present study, 53.8% of the dogs failed to complete the 20 min duration, which, given the submaximal test protocol, could suggest a reduced cardiorespiratory resilience. In contrast, the observed combination of lower lactate concentrations and heart rate, together with a less pronounced decrease in pCO_2_ and bicarbonate after therapy, can be interpreted as an improvement in cardiorespiratory resilience. Although the exercise protocol remained identical, these findings may indicate a shift toward predominantly aerobic energy metabolism and a reduced metabolic strain during exercise. Consequently, the compensatory decrease in pCO_2_ and bicarbonate was less pronounced.

The absence of statistically significant differences at timepoint 1 and 2 likely reflects that these measures were obtained under resting conditions. In particular, changes in pCO_2_ and HCO_3_^−^ should be interpreted cautiously, as these parameters reflect ventilation and acid–base regulation and cannot be considered direct measures of cardiorespiratory resilience. Because only the respiratory rate was recorded and the dogs were panting during measurements, tidal volume and minute ventilation were not quantified. Therefore, the extent to which the observed changes are attributable to altered ventilation versus other physiological or procedural factors cannot be determined conclusively. In addition, room humidity and patient-specific temperature correction were not assessed, which limits physiological interpretation beyond describing within-study changes.

Overall, the study results suggest that individualised weight therapy, which complies with current scientific recommendations, can be well monitored using new parameters in addition to the BCS in overweight dogs. In addition to the exclusion of relevant comorbidities and the creation of an individualised feeding plan, increasing the compliance of the pet owners is of particular importance. For this goal, the study paid particular attention to providing comprehensive information about the causes and consequences of obesity, as well as close, personal support. This holistic approach is considered crucial for successful treatment of obesity [9]. German (2016) draws similar conclusions, pointing out the high drop-out rate and the importance of intensive owner support in his retrospective analysis of weight reduction programmes [25].

The recorded change in the BCS in comparison to other weight measures confirms earlier studies on the clinical applicability and informative value of this scale. For example, a change in one point on the nine-point BCS scale corresponds approximately to a 10% change in weight, as Laflamme (1997) explains [13]. Our findings tend to support this assumption, as the mean change of about two BCS points is reflected in a mean change of about 15% weight loss. In addition, the work of German et al. (2006) shows that pet owners often underestimate the nutritional status of their dogs, a situation that can be significantly improved through targeted guidance and feedback [12]. This finding reinforces the decision to also systematically record the owners’ assessment in the present study and to correct it through professional feedback.

The changes in gait analysis in this study showed a significant decrease in absolute forces, an increase in vertical ground reaction forces (peak Fz, mean Fz, impulse Fz) normalised to body weight in almost all limbs, as well as a shift in load distribution towards the pelvic limbs and an improvement in the symmetry of the pelvic limbs. The observed shift in vertical impulse (Fz) towards the pelvic limbs after therapy suggests a change in load sharing between thoracic and pelvic limbs, consistent with a more caudal distribution of body mass and/or a reduction in relative forelimb overload. From a clinical perspective, reduced relative loading of the thoracic limbs may be relevant for dogs predisposed to or affected by thoracic-limb musculoskeletal disease (e.g., elbow or shoulder osteoarthritis), as the shift may indicate functional relief. This interpretation is in line with previous work describing associations between obesity, altered gait patterns, and orthopaedic disease and highlighting weight reduction as a therapeutic measure. In a review article by Marshall W. (2009) [31], the association between obesity and orthopaedic diseases, particularly osteoarthritis, was discussed, and weight reduction was highlighted as a therapeutic approach. The studies examined in the review utilised objective gait analysis to quantify weight loss-related alterations in gait patterns [31]. In the present study, significant changes in objective gait parameters were also detected during therapy. This finding indicates that the dogs included in this study may have benefited from weight reduction in a similar way to dogs with orthopaedic problems reported in the literature.

While ground reaction force changes indicate altered limb loading after weight loss, we did not quantify spatiotemporal or kinematic parameters (stride length, cadence, and stride-to-stride variability). Therefore, conclusions regarding improvement of the overall movement pattern or gait coordination cannot be drawn from the present data. The benefit of an objective gait analysis for evaluating the success of therapy in obesity is also underlined by the review article by Frye (2016), which also describes the connection between obesity and dysfunctional movement sequences [17]. Consistent with these findings, this study shows how cardiorespiratory parameters improve during therapy intervals. At the same time, the gait analysis revealed changes indicating a functional relief of the musculoskeletal system. The combination of fitness testing and gait analysis proved to be a practical tool for objectively recording functional improvements and could therefore be a valuable component of therapy evaluation.

While the present study demonstrates beneficial change in parameters during therapy intervals, potential challenges associated with weight reduction in dogs should be acknowledged. Inadequately controlled weight loss may be accompanied by undesired effects such as loss of lean body mass, insufficient nutrient intake, or changes in performance and behaviour. In this study, weight management was performed under close supervision and adapted to the individual needs of the dogs and their owners. The rate of weekly weight loss was regularly monitored, and dietary adjustments were made when necessary. All dietary rations were calculated and adjusted by veterinarians.

Despite the results, the present study has some limitations that should be taken into account. Firstly, the results were obtained in a single-arm, non-randomised trial without a control group. As the primary aim was to explore the change in the parameters in a real clinical setting, this study design is ethically justifiable compared with the inclusion of a non-treated control group. Yet, future studies should build on this approach, orienting on effect sizes and by implementing individual, yet standardised, diets and training protocols. Secondly, the loss of follow-up represents a limitation of this study, as attrition may introduce selection bias. Although baseline characteristics did not differ markedly between dogs that completed the programme and those that did not, this factor nevertheless limits the generalizability of the findings and should be considered when interpreting the results. A further limitation is the small sample size of 13 dogs, which could be re-examined and evaluated after completion of the therapy. This number of cases limits the generalizability of the results to the overall population of obese dogs. Yet, the study population is heterogeneous with different breeds, age groups, and initial conditions, which increases the suitability of the results for everyday clinical practice.

Concerning the gait analysis, data were obtained exclusively on an instrumented treadmill under controlled conditions. While this approach supports the standardisation and reproducibility of the measurements, it cannot be ruled out that the movement pattern on the treadmill deviates from the natural movement pattern in free space. In addition, the steps were selected by a single investigator without blinding, which may have an effect on the results. Furthermore, not re-assessing the preferred trotting speed after weight loss may represent a limitation, as weight reduction could alter a dog’s comfortable trotting velocity. Future studies should consider redetermining preferred speed at each time point and/or including statistical control for speed (e.g., covariate adjustment) to further strengthen causal interpretation. Likewise, the cooperation and motivation of the animals, both in the fitness test and in the gait analysis, may have influenced the results, although a habituation phase was included.

Despite limitations, the present results show the improvement in functional parameters of physical fitness in dogs during individualised therapy intervals. For follow-up studies, we recommend enrolling larger numbers of obese dogs using the standardised intervention protocols as outlined above. In particular, sampling should consider breed, age group, and exercise profiles to further specify weight reduction effects under therapy. While using a control group is ethically sensitive, cross-designs might be considered to assess the effect of different therapies on cardiorespiratory resilience and mobility over time. The inclusion of long-term data on the stability of the fitness improvements achieved after the end of therapy could also provide relevant information on the sustainability of the effects. In addition to cardiorespiratory and gait parameters, everyday activity data from a GPS tracker or pedometers could also be included for therapy monitoring in the future. A standardised assessment of quality of life from the owner’s perspective would also be of interest in order to supplement functional improvements with subjective components.

In summary, obesity represents a pressing and increasing animal welfare concern that would benefit from standardised assessment and evidence-based therapeutic approaches. The results of this study contribute to the establishment of objective measures for assessing cardiorespiratory resilience and mobility in obese dogs. These findings are consistent with previous studies and support the recommendation of novel objective parameters, such as cardiorespiratory resilience, for monitoring obesity in patients in routine clinical practice.

## 5. Conclusions

The present study elaborates on the change in cardiorespiratory and mobility parameters as indicators of physical fitness during individualised therapy intervals. In addition to reductions in body weight, the data demonstrate the potential of using a standardised submaximal fitness test and an instrumental gait analysis. The findings further suggest possible functional relief of the musculoskeletal system, as well as indications of increased exercise capacity after therapy. These findings suggest that generating individualised therapy plans for obesity in dogs should be increasingly monitored with parameters of systemic health and functional capacity that go beyond measures of BCS alone. Further studies are needed to systematically investigate the long-term effects in cross-designs with respect to breed-specific differences in therapy plans.

## Figures and Tables

**Table 1:** Change in weight and muscle parameters during therapy. (SP = Shapiro–Wilk, CI = confidence interval).

Variable	SP	S	Pr ≥ |S|	Mean	Cohens’ *d*	N	StdDev		
BCS	0.002	−45.5	0.0002	−2	−3.46	13	0.58		
MCS	0.0001	−5	0.125	−0.31	−0.64	13	0.48		
**Variable**	**SP**	**t-value**	***p*** **-value**	**Mean**	**Cohens’ *d***	**DF**	**StdDev**	**Lower CI**	**Upper CI**
Weigth (kg)	0.28	−6.17	<0.0001	−4.87	−1.71	12	2.84	−6.59	−3.15
Weight loss (%)	0.28	10.8	<0.0001	15.33	3	12	5.12	12.24	18.42

**Table 2:** Statistically significant parameters of cardiorespiratory resilience. (SP = Shapiro–Wilk, CI = confidence interval).

Variable	SP	t-Value	*p*-Value	Mean	Cohens’ *d*	DF	StdDev	Lower CI	Upper CI
HR (t3)	0.73	−3.52	<0.01	−26.46	−0.98	12	27.12	−42.85	−10.07
Lactate (t3)	0.34	−2.29	<0.05	−4.25	−0.634	12	6.71	−8.31	−0.199
PCO2 (t3)	0.45	2.26	<0.05	3.38	0.63	12	5.38	0.13	6.63
HCO_3_^−^ (t3)	0.98	2.55	<0.05	1.75	0.71	12	2.47	0.25	3.24
BE (t3)	0.49	2.05	0.063	0.92	0.57	12	1.62	−0.06	1.9

**Table 3:** Excerpt of statistically significant parameters of the linear mixed-effects model. (difference in parameter after and before therapy; “pl” = pelvic limb, “tl” = thoracic limb, “l” = left, “r” = right, CI = confidence interval). Model parameters are provided in Table S5.

Variable	t-Value	*p*-Value	Lower CI	Upper CI
IFz_pll	−7.28	<0.0001	−3.19	−1.83
IFz_plr	−7.69	<0.0001	−3.49	−2.06
IFz_tll	−8.19	<0.0001	−7.13	−4.36
IFz_tlr	−9.27	<0.0001	−8.53	−5.54
MFz_pll	−13.26	<0.0001	−13.71	−10.17
MFz_plr	−14.64	<0.0001	−15.22	−11.61
MFz_tll	−12.30	<0.0001	−26.41	−19.12
MFz_tlr	−12.71	<0.0001	−30.10	−22.02
PFz_pll	−10.52	<0.0001	−16.61	−11.37
PFz_plr	−9.51	<0.0001	−16.26	−10.68
PFz_tll	−9.78	<0.0001	−30.64	−20.36
PFz_tlr	−8.81	<0.0001	−29.99	−19.03

## Data Availability

The data presented in this study are available on request from the corresponding author.

## Supplementary Materials

The following supporting information can be downloaded at: https://www.mdpi.com/article/10.3390/ani16040678/s1, Table S1: Information about the individualized therapy diets; Table S2: Information on study population and the course of weight indicators; Table S3: Descriptives of variables; Table S4: Tests of cardiorespiratory parameters; Table S5: Model fit indicators (linear mixed-effect model) of gait parameters; Table S6: Estimates for Differences of variables in the linear mixed-effect models; Table S7: Mean load distribution changes.
