# Supplementary material for: Exploring Cardiorespiratory Resilience and Mobility as Indicators of Physical Fitness Under Individualised Therapy Intervals in Obese Dogs
Source: Animals (Basel). 2026 Feb 21;16(4):678. doi: 10.3390/ani16040678 (PMC12937215; doi:10.3390/ani16040678)
Supplement: Supplementary file 1 [file animals-16-00678-s001.zip › animals-4096797-supplementary.pdf]

Table S1. Information about the individualized therapy diets.

ID	Feeding practice (previous)	Feeding practice (treatment)	ME % of requirement (previous)	ME % of requirement (new)	protein % dry matter (previous)	protein % dry matter (new)	fat % dry matter (previous)	fat % dry matter (new)	fiber % dry matter (previous)	fiber % dry matter (new)
1	commercial dry diet	commercial weight loss dry diet	95	64	37.6	27.5	13	12.2	2.2	13.9
2	commercial dry diet	commercial weight loss dry diet	137	61	39.4	24.9	8.5	11	4.1	14
3	BARF	home cooked	71	59	46.2	43.1	34.2	16.4	2.8	6.8
4	commercial dry and wet diet	commercial weight loss dry diet	76	69	28.3	32.6	14.8	11.8	4.2	13
5	commercial dry diet	commercial dry and wet diet	102	69	30.5	27.3	7.9	7.9	7.7	16.1
6	home cooked	home cooked and commercial dry and wet diet	106	59	43.7	26.7	38.8	12.9	3.1	2.8
7	commercial dry and wet diet	commercial dry and wet diet	91	70	40	40.2	16.1	17.5	5.3	6.4
8	commercial dry diet	commercial dry diet	114	69	26.8	24.5	9.9	12.7	2.8	2
9	commercial wet diet	commercial dry and wet diet	124	69	28.9	27.8	15.7	12.8	0.9	11.7
10	commercial dry diet	home cooked	x	70	x	21.7	x	8.7	x	3.8
11	home cooked and commercial dry diet	commercial dry and wet diet	94	70	30.9	35	8.8	14.4	11.6	9.5
12	BARF and commercial dry diet	home cooked and commercial dry diet	119	70	26	25.3	19.8	20	4.9	5.4
13	commercial dry diet	commercial dry diet	x	70	x	31	x	15.4	x	3.5

Table S2. Information on study population and the course of weight indicators. BCS = Body Condition Score, MCS = Muscle Condition Score.

ID	Age in years	sex	Breed	Weight (before)	Weight (after)	Weight loss	BCS (before)	BCS (after)	MCS (before)	MCS (after)
**1**	10	Neutered female	Beagle	11.4 kg	10.4 kg	12,3%	8	7	Mild loss	Normal
**2**	8	Neutered male	Labrador Retriever	44.6 kg	35.4 kg	20,6%	8	5	Normal	Normal
**3**	7	Female	Australian Shepherd	25.2 kg	22.6 kg	10,3%	7	5	Normal	Normal
**4**	5	Female	Australian Shepherd	29.6 kg	24.4 kg	17,6%	7	5	Normal	Normal
**5**	10	Neutered male	Labrador Retriever	41.8 kg	33.6 kg	19,6%	8	6	Mild loss	Normal
**6**	4	Female	Labrador Retriever	37.8 kg	34.0 kg	10,1%	8	6	Normal	Normal
**7**	9	Neutered female	Mixed breed	24.6 kg	19.4 kg	21,1%	7	5	Mild loss	Normal
**8**	5	Neutered female	Mixed breed	13.0 kg	11.1 kg	14,6%	7	5	Normal	Normal
**9**	5	Neutered male	Miniature American Shepherd	17.8 kg	14.6 kg	18,0%	8	5	Normal	Normal
**10**	6	Neutered male	German Shepherd	41.8 kg	37.0 kg	11,5%	7	5	Normal	Normal
**11**	10	Neutered female	Australian Shepherd	22.8 kg	19.8 kg	13,2%	7	6	Normal	Normal
**12**	4	Male	Labrador Retriever	44.8 kg	34.4 kg	23,2%	7	5	Normal	Normal
**13**	8	Neutered female	Greater Swiss Mountain Dog	49.0 kg	44.2 kg	9.8%	7	5	Moderate loss	Mild loss

Table S3. Descriptives of variables

Variable	N	Mean	Std Dev	Median	Minimum	Maximum
age	13	7.00	2.31	7.00	4.00	10.00
BCS_before	13	7.39	0.51	7.00	7.00	8.00
BCS_after	13	5.39	0.65	5.00	5.00	7.00
BE_t1_after	13	-2.05	2.63	-2.70	-8.500	1.70
BE_t1_before	13	-2.53	1.12	-2.60	-4.00	-0.70
BE_t2_after	13	-2.14	1.03	-2.20	-4.100	-0.20
BE_t2_before	13	-2.50	1.42	-2.10	-4.900	-0.30
BE_t3_after	13	-2.22	1.80	-1.40	-6.600	0.10
BE_t3_before	13	-3.15	1.51	-3.70	-5.200	-0.80
HCO_3^-_t1_after	13	21.77	3.39	21.60	13.300	27.40
HCO_3^-_t1_before	13	21.04	1.29	20.90	18.900	22.90
HCO_3^-_t2_after	13	20.66	1.75	20.70	17.00	23.40
HCO_3^-_t2_before	13	19.79	1.49	19.60	17.400	22.60
HCO_3^-_t3_after	13	19.66	2.40	20.40	14.600	23.90
HCO_3^-_t3_before	13	17.92	1.84	18.30	14.100	20.90
Weight_before	13	31.09	12.93	29.60	11.400	49.00
Weight_after	13	26.22	10.89	24.40	10.400	44.20
Weight_kg_before	13	31.09	12.93	29.60	11.400	49.00
Weight_kg_after	13	26.22	10.89	24.40	10.400	44.20
HR_t1_after	13	95.69	25.26	90.00	60.00	146.00
HR_t1_before	13	97.08	31.36	96.00	60.00	190.00
HR_t2_after	13	94.15	19.31	90.00	60.00	124.00
HR_t2_before	13	105.54	30.27	100.00	68.00	190.00
HR_t3_after	13	110.92	23.06	104.00	76.00	168.00
HR_t3_before	13	137.39	25.40	138.00	108.00	190.00
IFzpll%_before	13	7.93	2.53	7.56	4.73	15.30
IFzpll%_after	13	8.85	2.32	8.36	5.42	14.98
IFzpll_before	13	26.26	18.28	21.96	6.83	73.53
IFzpll_after	13	24.25	15.60	24.81	6.90	64.95
IFzplr%_before	13	7.97	2.34	7.50	5.87	14.92
IFzplr%_after	13	8.72	2.21	8.37	5.04	14.24
IFzplr_before	13	26.19	17.70	21.78	7.49	71.71
IFzplr_after	13	23.92	15.04	24.82	6.74	61.72
IFztll%_before	13	12.57	2.52	12.57	8.97	17.27
IFztll%_after	13	13.30	2.36	13.00	9.96	16.74
IFztll_before	13	40.74	22.37	36.50	11.12	70.81
IFztll_after	13	35.69	17.80	37.73	10.17	56.38
IFztlr%_before	13	12.73	2.97	12.61	8.84	18.74
IFztlr%_after	13	13.21	2.50	13.11	9.22	17.35
IFztlr_before	13	41.81	24.20	36.62	10.05	76.84
IFztlr_after	13	35.43	17.59	35.44	9.41	57.20
Lac_t1_after	13	13.19	4.45	12.00	8.50	23.30
Lac_t1_before	13	13.18	4.41	13.00	7.20	23.20
Lac_t2_after	13	13.32	3.07	12.80	8.60	20.10
Lac_t2_before	13	14.52	3.02	15.00	8.30	19.50
Lac_t3_after	13	16.00	5.70	14.30	8.80	26.50
Lac_t3_before	13	20.25	6.48	20.70	8.80	29.90
MCS_before	13	1.39	0.65	1.00	1.00	3.00
MCS_after	13	1.08	0.28	1.00	1.00	2.00
MFzpll%_before	13	32.95	5.79	32.76	19.72	44.23
MFzpll%_after	13	34.69	5.32	35.73	24.65	43.18
MFzpll_before	13	98.42	39.86	117.18	43.78	143.18
MFzpll_after	13	86.45	31.47	102.19	38.35	126.02
MFzplr%_before	13	33.45	6.00	33.20	26.57	45.19
MFzplr%_after	13	34.35	5.53	34.67	22.90	43.21
MFzplr_before	13	99.20	38.66	106.33	42.77	145.26
MFzplr_after	13	85.78	31.67	103.42	37.44	119.47

MFztll%_before	13	52.72	7.17	56.36	36.07	59.86
MFztll%_after	13	54.58	7.09	55.51	36.12	64.33
MFztll_before	13	160.19	70.21	173.39	63.56	258.99
MFztll_after	13	137.69	53.23	153.98	56.47	204.75
MFztlr%_before	13	53.04	7.79	55.82	37.72	62.67
MFztlr%_after	13	54.10	7.97	55.97	33.74	63.54
MFztlr_before	13	162.64	74.83	176.89	62.43	267.56
MFztlr_after	13	136.72	53.66	146.31	52.26	204.89
PCO2_t1_after	13	34.92	6.41	34.90	20.80	46.80
PCO2_t1_before	13	33.51	4.10	34.30	27.80	41.10
PCO2_t2_after	13	30.62	4.75	31.00	22.60	39.80
PCO2_t2_before	13	28.33	2.95	27.90	23.80	34.60
PCO2_t3_after	13	27.26	5.11	26.20	20.80	40.30
PCO2_t3_before	13	23.89	4.88	25.50	14.90	30.70
PFzpll%_before	13	54.76	10.93	56.01	31.79	72.23
PFzpll%_after	13	60.19	11.90	62.89	37.24	77.76
PFzpll_before	13	162.74	67.15	174.63	72.94	240.61
PFzpll_after	13	148.71	54.91	161.45	71.58	220.72
PFzplr%_before	13	55.44	10.90	57.02	36.82	76.72
PFzplr%_after	13	60.91	10.48	62.06	37.33	79.31
PFzplr_before	13	164.29	66.16	176.99	75.89	239.27
PFzplr_after	13	150.82	54.62	161.85	67.09	219.25
PFztll%_before	13	85.24	13.97	87.40	57.37	105.15
PFztll%_after	13	93.07	15.04	94.50	53.73	111.65
PFztll_before	13	260.97	121.15	275.79	102.97	425.28
PFztll_after	13	235.54	97.89	232.97	95.37	367.35
PFztlr%_before	13	85.73	14.70	87.69	58.80	104.90
PFztlr%_after	13	94.21	17.18	95.16	54.09	123.02
PFztlr_before	13	263.85	125.45	282.64	100.28	431.95
PFztlr_after	13	239.33	101.63	234.53	87.54	377.15
pH_t1_after	13	7.41	0.03	7.42	7.37	7.45
pH_t1_before	13	7.42	0.04	7.410	7.35	7.49
pH_t2_after	13	7.45	0.038	7.46	7.39	7.53
pH_t2_before	13	7.46	0.031	7.46	7.42	7.52
pH_t3_after	13	7.48	0.042	7.47	7.39	7.55
pH_t3_before	13	7.50	0.07	7.51	7.42	7.62
PO2_t1_after	13	47.94	29.47	38.10	26.50	140.20
PO2_t1_before	13	40.01	6.87	39.60	29.50	53.10
PO2_t2_after	13	44.05	10.66	43.60	27.80	65.50
PO2_t2_before	13	47.45	11.13	45.20	34.70	73.00
PO2_t3_after	13	60.50	23.42	52.00	39.50	109.40
PO2_t3_before	13	58.06	12.97	54.1	39.9	90.1

Table S4. Tests of cardiorespiratory parameters. (CI= Confidence Interval)

Variables	Shapiro-Wilk	t-value	Probt	Mean	StdDev	Lower CI	Upper CI	Cohens' *d*	DF
BE_t1_before-BE_t1_after	0,91	0,62	0,5480	0,48	2,83	-1,22	2,19	0,17	12
BE_t2_before-BE_t2_after	0,35	0,93	0,3699	0,36	1,40	-0,48	1,21	0,26	12
BE_t3_before-BE_t3_after	0,49	2,05	0,0631	0,92	1,62	-0,06	1,90	0,57	12
HCO_3^-_t1_before-HCO_3^-_t1_after	0,09	0,81	0,4344	0,73	3,26	-1,24	2,70	0,22	12
HCO_3^-_t2_before-HCO_3^-_t2_after	0,24	1,64	0,1264	0,87	1,91	-0,28	2,02	0,46	12
HCO_3^-_t3_before-HCO_3^-_t3_after	0,98	2,55	0,0256	1,75	2,47	0,25	3,24	0,71	12
HR_t1_before-HR_t1_after	0,30	-0,28	0,7878	-1,38	18,14	-12,34	9,58	-0,08	12
HR_t2_before-HR_t2_after	0,44	-1,64	0,1271	-11,38	25,04	-26,52	3,75	-0,45	12
HR_t3_before-HR_t3_after	0,73	-3,52	0,0042	-26,46	27,12	-42,85	-10,07	-0,98	12
Lac_t1_before-Lac_t1_after	0,08	0,01	0,9950	0,01	4,31	-2,60	2,61	0,00	12
Lac_t2_before-Lac_t2_after	0,65	-1,16	0,2688	-1,20	3,73	-3,45	1,05	-0,32	12
Lac_t3_before-Lac_t3_after	0,34	-2,29	0,0413	-4,25	6,71	-8,31	-0,20	-0,63	12
PCO2_t1_before-PCO2_t1_after	0,23	0,82	0,4255	1,41	6,15	-2,31	5,13	0,23	12
PCO2_t2_before-PCO2_t2_after	0,73	1,82	0,0944	2,29	4,55	-0,46	5,04	0,50	12
PCO2_t3_before-PCO2_t3_after	0,45	2,26	0,0429	3,38	5,38	0,13	6,63	0,63	12
PO2_t2_before-PO2_t2_after	0,93	-0,67	0,5149	-3,43	18,43	-14,57	7,71	-0,19	12
PO2_t3_before-PO2_t3_after	0,27	0,44	0,6688	2,45	20,12	-9,71	14,60	0,12	12
pH_t1_before-pH_t1_after	0,06	-0,29	0,7785	0,00	0,05	-0,03	0,02	-0,08	12
pH_t2_before-pH_t2_after	0,76	-1,20	0,2522	-0,01	0,04	-0,04	0,01	-0,33	12
pH_t3_before-pH_t3_after	0,35	-1,60	0,1366	-0,02	0,05	-0,05	0,01	-0,44	12

Variable	Shapiro-Wilk	S	Pr >= |S|	Mean	StdDev	Cohens' *d*	N
PO2_t1_before-PO2_t1_after	0,00	10,50	0,4973	7,93	26,22	0,30	13

Table S5. Model fit indicators (linear mixed-effect model) of gait parameters. (CI= Confidence Interval, effect: time after therapy= reference)

Variable	NumDf	DenDf	F-value	Prob. F	AIC	Effect	Estimate	StdErr	DF	t-value	Probt	Ratio (random / total residuals)
IFz_pll%	1,00	246,00	61,90	<0.0001	685,15	Intercept	8,71	0,66	12,13	13,24	<0.0001	0,90
						time (before therapy)	-0,77	0,10	246,00	-7,87	<0.0001	
IFz_pll	1,00	246,00	53,06	<0.0001	1352,44	Intercept	23,76	4,64	12,03	5,12	<0.001	0,97
						time (before therapy)	2,51	0,34	246,00	7,28	<0.0001	
IFz_plr%	1,00	246,00	33,76	<0.0001	712,70	Intercept	8,58	0,62	12,17	13,94	<0.0001	0,87
						time (before therapy)	-0,60	0,10	246,00	-5,81	<0.0001	
IFz_plr	1,00	246,00	59,12	<0.0001	1374,40	Intercept	23,44	4,50	12,04	5,21	<0.001	0,97
						time (before therapy)	2,78	0,36	246,00	7,69	<0.0001	
IFz_tll%	1,00	246,00	10,49	<.0.01	940,94	Intercept	13,14	0,65	12,40	20,27	<0.0001	0,75
						time (before therapy)	-0,53	0,16	246,00	-3,24	<0.01	
IFz_tll	1,00	246,00	67,11	<0.0001	1705,72	Intercept	35,12	5,46	12,10	6,43	<0.0001	0,92
						time (before therapy)	5,75	0,70	246,00	8,19	<0.0001	
IFz_tlr%	1,00	246,00	2,57	0,11	967,90	Intercept	13,02	0,73	12,34	17,87	<0.0001	0,78
						time (before therapy)	-0,28	0,17	246,00	-1,60	0,11	
IFz_tlr	1,00	246,00	86,00	<0.0001	1745,47	Intercept	34,82	5,70	12,11	6,11	<0.0001	0,92
						time (before therapy)	7,04	0,76	246,00	9,27	<0.0001	
MFz_pll%	1,00	246,00	32,11	<0.0001	1273,17	Intercept	34,71	1,50	12,26	23,07	<0.0001	0,82
						time (before therapy)	-1,76	0,31	246,00	-5,67	<0.0001	
MFz_pll	1,00	246,00	175,88	<0.0001	1842,82	Intercept	86,47	9,86	12,05	8,77	<0.0001	0,96
						time (before therapy)	11,94	0,90	246,00	13,26	<0.0001	
MFz_plr%	1,00	246,00	6,57	<0.05	1334,71	Intercept	34,35	1,50	12,34	22,95	<0.0001	0,78
						time (before therapy)	-0,90	0,35	246,00	-2,56	<0.05	
MFz_plr	1,00	246,00	214,46	<0.0001	1851,01	Intercept	85,78	9,69	12,05	8,85	<0.0001	0,96
						time (before therapy)	13,42	0,92	246,00	14,64	<0.0001	
MFz_tll%	1,00	246,00	16,92	<0.001	1434,13	Intercept	54,58	1,89	12,31	28,88	<0.0001	0,79
						time (before therapy)	-1,75	0,43	246,00	-4,11	<0.0001	
MFz_tll	1,00	246,00	151,40	<0.0001	2210,12	Intercept	137,69	16,91	12,07	8,14	<0.0001	0,94
						time (before therapy)	22,77	1,85	246,00	12,30	<0.0001	

MFz_tlr%	1,00	246,00	3,40	0,07	1508,85	Intercept	53,96	2,03	12,36	26,57	<0.0001	0,77
						time (before therapy)	-0,91	0,49	246,00	-1,84	0,07	
MFz_tlr	1,00	246,00	161,42	<0.0001	2261,87	Intercept	136,59	17,62	12,08	7,75	<0.0001	0,94
						time (before therapy)	26,06	2,05	246,00	12,71	<0.0001	
PFz_pll%	1,00	246,00	134,83	<0.0001	1494,25	Intercept	60,19	3,11	12,14	19,38	<0.0001	0,90
						time (before therapy)	-5,45	0,47	246,00	-11,61	<0.0001	
PFz_pll	1,00	246,00	110,65	<0.0001	2047,68	Intercept	148,71	16,88	12,04	8,81	<0.0001	0,97
						time (before therapy)	13,99	1,33	246,00	10,52	<0.0001	
PFz_plr%	1,00	246,00	100,49	<0.0001	1566,72	Intercept	60,91	2,85	12,22	21,34	<0.0001	0,84
						time (before therapy)	-5,47	0,55	246,00	-10,02	<0.0001	
PFz_plr	1,00	246,00	90,49	<0.0001	2078,32	Intercept	150,82	16,69	12,04	9,04	<0.0001	0,97
						time (before therapy)	13,47	1,42	246,00	9,51	<0.0001	
PFz_tll%	1,00	246,00	120,86	<0.0001	1703,26	Intercept	93,05	3,87	12,20	24,02	<0.0001	0,85
						time (before therapy)	-7,80	0,71	246,00	-10,99	<0.0001	
PFz_tll	1,00	246,00	95,60	<0.0001	2392,88	Intercept	235,47	30,14	12,05	7,81	<0.0001	0,96
						time (before therapy)	25,50	2,61	246,00	9,78	<0.0001	
PFz_tlr%	1,00	246,00	113,68	<0.0001	1761,54	Intercept	94,21	4,19	12,22	22,46	<0.0001	0,85
						time (before therapy)	-8,49	0,80	246,00	-10,66	<0.0001	
PFz_tlr%	1,00	246,00	77,56	<0.0001	2425,69	Intercept	239,33	31,19	12,05	7,67	<0.0001	0,96
						time (before therapy)	24,51	2,78	246,00	8,81	<0.0001	

Table S6. Estimates for Differences of variables in the linear mixed-effect models. (CI=Confidence Interval, IFz (N·s), MFz (N), PFz (N), Lactate (mmol/l), PCO2 (mmHg), PO2 (mmHg), HCO_3^- (mmol/l), HR (beats/min), pl + pelvic limbs, tl = Thoracic limbs, l = left, r = right)

Variable	Difference	Estimate	StdDev	DF	t-value	p-value	Lower CI	Upper CI
IFz_pll%	after – before (therapy)	0,77	0,10	246,00	7,87	<0.0001	0,58	0,96
IFz_pll	after - before	-2,51	0,34	246,00	-7,28	<0.0001	-3,19	-1,83
IFz_plr%	after - before	0,60	0,10	246,00	5,81	<0.0001	0,40	0,81
IFz_plr	after - before	-2,78	0,36	246,00	-7,69	<0.0001	-3,49	-2,06
IFz_tll%	after - before	0,53	0,16	246,00	3,24	<.0.01	0,21	0,86

IFz_tll	after - before	-5,75	0,70	246,00	-8,19	<0.0001	-7,13	-4,36
IFz_tlr%	after - before	0,28	0,17	246,00	1,60	0,110	-0,06	0,62
IFz_tlr	after - before	-7,04	0,76	246,00	-9,27	<0.0001	-8,53	-5,54
MFz_pll%	after - before	1,76	0,31	246,00	5,67	<0.0001	1,15	2,37
MFz_pll	after - before	-11,94	0,90	246,00	-13,26	<0.0001	-13,71	-10,17
MFz_plr%	after - before	0,90	0,35	246,00	2,56	0,011	0,21	1,59
MFz_plr	after - before	-13,42	0,92	246,00	-14,64	<0.0001	-15,22	-11,61
MFz_tll%	after - before	1,75	0,43	246,00	4,11	0,000	0,91	2,59
MFz_tll	after - before	-22,77	1,85	246,00	-12,30	<0.0001	-26,41	-19,12
MFz_tlr%	after - before	0,91	0,49	246,00	1,84	0,066	-0,06	1,88
MFz_tlr	after - before	-26,06	2,05	246,00	-12,71	<0.0001	-30,10	-22,02
PFz_pll%	after - before	5,45	0,47	246,00	11,61	<0.0001	4,52	6,37
PFz_pll	after - before	-13,99	1,33	246,00	-10,52	<0.0001	-16,61	-11,37
PFz_plr%	after - before	5,47	0,55	246,00	10,02	<0.0001	4,40	6,55
PFz_plr	after - before	-13,47	1,42	246,00	-9,51	<0.0001	-16,26	-10,68
PFz_tll%	after - before	7,80	0,71	246,00	10,99	<0.0001	6,40	9,20
PFz_tll	after - before	-25,50	2,61	246,00	-9,78	<0.0001	-30,64	-20,36
PFz_tlr%	after - before	8,49	0,80	246,00	10,66	<0.0001	6,92	10,05
PFz_tlr%	after - before	-24,51	2,78	246,00	-8,81	<0.0001	-29,99	-19,03

Table S7. Mean load distribution changes.

	Thoracic limb		Pelvic limb		Load Distribution	Ratio
	**left**	**right**	**left**	**right**	**tl/pl**	LD_{before} / LD_{after}
Ifz						
before	40.74	41.81	26.26	26.19	29.33	1.09
after	35.69	35.43	24.25	23.92	26.85	
MFz						
before	160.19	162.64	98.42	99.2	102.48	1.15

	after	137.69	136.72	86.45	85.78	88.95	
PFz							
	before	260.97	263.85	162.74	164.29	167.51	1.09
	after	235.54	239.33	148.71	150.82	154.01	
